# The Role of Seminal Oxidative Stress Scavenging System in the Pathogenesis of Sperm DNA Damage in Men Exposed and Not Exposed to Genital Heat Stress

**DOI:** 10.3390/ijerph19052713

**Published:** 2022-02-25

**Authors:** Monika Fraczek, Angelika Lewandowska, Marta Budzinska, Marzena Kamieniczna, Lukasz Wojnar, Kamil Gill, Malgorzata Piasecka, Michal Kups, Anna Havrylyuk, Valentina Chopyak, Jozef Nakonechnyy, Andrij Nakonechnyy, Maciej Kurpisz

**Affiliations:** 1 Institute of Human Genetics, Polish Academy of Sciences, 60-479 Poznan, Poland; angelika878@wp.pl (A.L.); marta.budzinska@igcz.poznan.pl (M.B.); marzena.kamieniczna@igcz.poznan.pl (M.K.); 2 Clinic of Urology and Oncological Urology, Poznan University of Medical Sciences, 61-285 Poznan, Poland; lukaszwojnar@gmail.com; 3 Department of Histology and Developmental Biology, Pomeranian Medical University in Szczecin, 71-210 Szczecin, Poland; kamilgill@wp.pl (K.G.); mpiasecka@ipartner.com.pl (M.P.); 4 Department and Clinic Urology and Oncological Urology, Regional Specialist Hospital in Szczecin, 71-455 Szczecin, Poland; michalkups1@gmail.com; 5 The Fertility Partnership Vitrolive in Szczecin, 70-483 Szczecin, Poland; 6 Department of Clinical Immunology and Allergology, Danylo Halytskyy Lviv National Medical University, 79008 Lviv, Ukraine; ahavrylyuk@meta.ua (A.H.); chopyakv@ukr.net (V.C.); 7 Department of Urology, Danylo Halytskyy Lviv National Medical University, 79010 Lviv, Ukraine; nyosyf@ukr.net; 8 Department of Paediatric Surgery, Danylo Halytskyy Lviv National Medical University, 79059 Lviv, Ukraine; andrurol@gmail.com

**Keywords:** genital heat stress, oxidative stress scavenging system, sperm DNA damage

## Abstract

Responding to the need for the verification of some experimental animal studies showing the involvement of oxidative stress in germ cell damage in the heat-induced testis, we investigated the possibility of a direct relationship between seminal oxidative stress markers (total antioxidant capacity, catalase activity, superoxide dismutase activity, and malondialdehyde concentration) and ejaculated sperm chromatin/DNA integrity (DNA fragmentation and chromatin condensation abnormalities) in distinct groups of men exposed and not exposed to prolonged scrotal hyperthermia. A statistical increase in the proportion of sperm with DNA fragmentation was observed in all the studied subgroups compared to the fertile men. In turn, the groups subjected to heat stress as professional drivers or infertile men with varicocele presented greater disturbances in the oxidative stress scavenging system than men not exposed to genital heat stress. Based on the comparative analysis of the studied parameters, we can conclude that alterations in the seminal oxidative stress scavenging system are directly engaged in the pathogenesis of ejaculated sperm DNA damage regardless of the intensity of the impact of thermal insult. To the best of our knowledge, this study, for the first time, revealed the co-existence of oxidative stress and sperm DNA damage in the semen of professional drivers.

## 1. Introduction

It is well established that the production and maturation of mammalian sperm are highly dependent on the scrotal temperature and run efficiently at a minimum of 2 °C below that of the body core. In this context, the dysfunction of local thermoregulatory systems is considered to be one of the main risk factors for male subfertility/infertility [1]. There are many different factors and situations associated with a raised scrotal temperature that induce genital heat stress. Everyday behavior such as a sedentary lifestyle, hot bath or sauna, tight clothing, or occupations performed in high temperatures are examples of many environmental factors provoking hyperthermia in male gonads. In addition, clinical factors resulting from pathophysiological conditions such as fever, obesity, cryptorchidism, and varicocele are involved in the scrotal temperature increase [2,3].

One of the critical mechanisms by which heat stress induces testicular disorders is the oxidative stress pathway. The excessive generation of reactive oxygen species (ROS) in mitochondria, depolarization of mitochondrial membrane potential, changes in cell membrane fluidity and stability, production of heat shock protein (HSP), impairment of DNA synthesis, and changes in gene expression and signal transduction are just some of the molecular effects of heat stress occurring in male germ cells [4]. The majority of experimental animal studies reported heat-induced oxidative stress responses in male gonads, such as apoptosis of the germ cells, cell cycle arrest, and/or disturbances in the oxidative stress scavenging system [5,6,7,8,9]. Although the aforementioned effects of the oxidative stress in heat-induced injury in the testes have been confirmed many times, the involvement of the thermogenic factor in the pathomechanism of the ejaculated sperm oxidation still presents a puzzle.

The destructive effect of oxidative stress on male gametes is mainly associated with the peroxidative processes of sperm membrane lipids, proteins, impairment of mitochondrial membrane potential, and DNA fragmentation [10,11,12]. Regarding the results of ROS interactions with sperm subcellular structures, variations primarily concern the DNA integrity. There is a generally accepted view that three different pathogenic mechanisms, including incomplete apoptosis in the testis, alterations in the sperm maturation process, and free radical attack, may contribute to the DNA damage in ejaculated sperm cells [13]. However, there is increasing evidence that the majority of sperm DNA breaks is of oxidative origin, especially in the context of a theory of intrinsic mitochondrial-dependent apoptosis in mature spermatozoa initiated by the increased ROS production in the mitochondrial chain [13,14]. The increase in the percentage of sperm-fragmented DNA has often been reported in ejaculates of men with varicocele [15,16,17,18]. However, the adverse effects of cryptorchidism [19], obesity [20], transient scrotal hyperthermia [21], and a sedentary lifestyle [22] on sperm nuclear DNA have also been demonstrated.

In light of the data mentioned above, there is a tendency to combine oxidative stress and sperm death processes in the pathomechanism of male infertility [23,24]. It has been widely postulated, especially in the case of varicocele, to be the most common pathology that may be fundamental to causing male infertility. However, the involvement of the thermogenic factor in varicocele patients has not been well estimated. The present work addresses a link between seminal oxidative stress and sperm chromatin/DNA status including chromatin stability, condensation, and DNA fragmentation in males exposed to scrotal hyperthermia of both internal (varicocele) and external (professional drivers) origin, as well as those not exposed to prolonged genital heat stress. The study aimed to examine the clinical verification of some current concepts regarding possible pathways that can be involved in heat-induced sperm chromatin/DNA damage. It should be noted here that this work is a continuation of our previously published clinical retrospective study in which the associations among standard semen quality, seminal oxidative stress, and biochemical status in the context of genital heat stress were reported [25].

## 2. Materials and Methods

### 2.1. Study Population

This retrospective study continues the previously published original study from a multi-center research project [25]. The study protocol received approval from the Local Bioethical Committee at the Poznan University of Medical Sciences, Poland, following relevant guidelines and regulations. All individual participants were informed of the study design and provided informed consent for the research on donated samples. To perform this study, 232 men aged 23–40 years were enrolled. The subjects were recruited from the Andrology Outpatient Clinics in Poznan, Szczecin, and Lviv and via traditional and social media advertising. A detailed medical history was scrutinized, while andrological and ultrasound examinations to confirm or exclude the presence of varicocele were performed. Additionally, the men completed a questionnaire containing questions on their general health, genitourinary and systemic diseases, working conditions, and lifestyle. The main exclusion criteria applied were: age over 40 years, body mass index ≥ 30, tobacco smoking, co-existence of systemic or locally active inflammations, history of cryptorchidism, and exposure to double local temperature factor. The subjects were categorized according to their medical history and survey data and contained: (A) a group of healthy fertile men not exposed to prolonged genital heat stress, which served as the control group (*n* = 30); (B) a group of professional drivers; minimum 2 years in the profession (*n* = 61); (C) a group of infertile men with varicocele (*n* = 101), and (D) a group of infertile men not exposed to prolonged genital heat stress (*n* = 40). Subjects were classified as fertile when they had fathered at least one child over the past two years and infertile when they had failed to achieve natural conception for at least 12 months of unprotected sexual intercourses without any apparent reason on the partner’s side. The varicoceles were diagnosed when the dilation of the vessels of the pampiniform plexus was ≥3 mm in diameter.

### 2.2. Semen Sample Preparation

All semen samples were obtained by masturbation after 3–5 days of sexual abstinence before medical treatment (surgical interventions, medicines, and supplements). The samples were examined after liquefaction at room temperature for 30 min [25]. Spermatozoa from collected samples were separated from seminal plasma by centrifugation at 1800 rpm for 7 min. Seminal plasma was centrifuged again at 3500 rpm for 5 min, then divided into aliquots and stored at −75 °C for the determination of oxidative stress parameters. Sperm pellets were washed in phosphate-buffered saline (PBS) at 1800 rpm for 7 min. An aliquot of sperm suspension was used for detecting the SCSA by flow cytometry. Sperm smears were also prepared. They were fixed in 3% glutaraldehyde at room temperature for 30 min and stored at −20 °C to assess sperm chromatin maturity in the aniline blue (AB) test. The remaining part of sperm pellets was fixed in 1% formaldehyde at 4 °C for 20 min. To avoid sperm sticking, the cells were transferred to a fixative agent and mixed vigorously. After two washes in PBS, the fixed sperm cells were resuspended in cold 75% ethanol and stored at −20 °C to assess sperm DNA fragmentation in the TUNEL assay. Seminal plasma and fixed spermatozoa collected in Szczecin and Lviv were transported on dry ice to the Andrology Laboratory in Poznan. All the samples were stored for a maximum of 3 months before being analyzed.

### 2.3. Determination of Oxidative Stress Parameters in Seminal Plasma

The levels of seminal antioxidant parameters such as total antioxidant capacity (TAC), and catalase and superoxide dismutase (SOD) activity were determined with commercially available kits from Cayman Chemical (Cayman Chemical, Ann Arbor, MI, USA), while the OXISelect TBARS Assay Kit (Cell Biolabs Inc., San Diego, CA, USA) was used for malondialdehyde (MDA) quantification in seminal plasma.

#### 2.3.1. Total Antioxidant Capacity (TAC)

The reaction mixture contained: 150 µL of chromogen (ABTS^®^), 10 µL of diluted seminal plasma (1:9) or a suitable standard, 10 µL of metmyoglobin, and 40 µL of H_2_O_2_. The samples were incubated with shaking for 5 min at room temperature. After incubation, suppression of the absorbance of blue-green ABTS^®+^ was measured at 405 nm in a spectrophotometric microplate reader (ELx808, Bio Tek Instruments, Inc. Winooski, VT, USA). The results were reported as µM of Trolox equivalent. Each sample was determined in duplicate [26].

#### 2.3.2. Catalase Activity

The reaction mixture contained: 100 µL of Assay Buffer, 20 µL of seminal plasma or a suitable standard, 30 µL of methanol, and 20 µL of H_2_O_2_. After a 20 min incubation with shaking at room temperature, the reaction was stopped by adding 30 µL of sodium hydroxide. Then, 30 µL of chromogen (catalase purpald) was added, and the samples were incubated again (constant shaking) for 10 min at room temperature. After incubation, 10 µL of potassium periodate was added and the samples were incubated for another 5 min with shaking at room temperature. The intensity of purple color was measured at 540 nm in a spectrophotometric microplate reader (ELx808). Formaldehyde content was calculated based on the linear regression curve for standard samples. The results were finally expressed as nM/min/mL. Each sample was determined in duplicate [27].

#### 2.3.3. Superoxide Dismutase (SOD) Activity

The reaction mixture contained: 200 µL of tetrazolium salt, 10 µL of xanthine oxidase, and 10 µL of diluted seminal plasma (1:2) or a suitable standard. After a 30 min incubation with shaking at room temperature, the absorbance of the samples was measured at 450 nm in a spectrophotometric microplate reader (ELx808). The results were calculated based on the linear regression curve for standard samples and finally expressed as U/mL. Each sample was determined in duplicate [28].

#### 2.3.4. Malondialdehyde (MDA) Concentration

The reaction mixture contained: 100 µL of seminal plasma or equivalent standard, 100 µL of SDS lysis buffer, and 250 µL of TBA solution. After a 45 min incubation in a water bath at 95 °C, the reaction was stopped by cooling on ice. The samples were centrifuged at 3000 rpm for 15 min. The supernatant obtained was extracted with n-butanol (1:1, *v*/*v*). For this purpose, the samples were vortexed for 2 min and centrifuged at 10,000× g for 5 min. The butanol fraction was transferred to a 96-well microplate and the absorbance at 532 nm was measured in a spectrophotometric microplate reader (ELx808). The MDA content in seminal plasma was read directly from the MDA standard curve. Water served as a blank control sample. The results were expressed as µM/mL. Each sample was determined in duplicate [29].

### 2.4. Determination of Sperm Nuclear DNA Integrity

#### 2.4.1. Flow Cytometry Measurements and Data Analysis

A Beckman Coulter flow cytometer (Cell Lab Quanta SC MPL, Beckman Coulter, Fullerton, CA, USA) equipped with an argon laser with a wavelength of 488 nm was used in the study. The sperm population was gated based on signals from Electronic Volume (EV, parameter depending on cells size) and Side Scatter (SS, parameter depending on cells granularity) detectors. A total of 10,000 events were analyzed at a flow rate of 200–250 cells per second in each sample. The intensity of green fluorescence (480–550 nm) was measured in the fluorescence channel FL1, while the power of red fluorescence (560–670 nm) was measured in channel FL3. Signals from SS and EV detectors were recorded on a linear scale and from FL1 and FL3 on a logarithmic scale. Fluorescence measurements were repeated two times with distinct samples. The data obtained were analyzed using the Cell Lab Quanta S.C. MPL Analysis software (Beckman Coulter, Brea, CA, USA).

#### 2.4.2. Sperm Chromatin Structure Assay (SCSA)

The SCSA test was applied to assess the susceptibility of sperm DNA to acid-induced DNA denaturation, followed by staining with acridine orange, a metachromatic fluorescent dye. Acridine orange emits green fluorescence when it binds to double-stranded DNA and red fluorescence when it binds to single-stranded DNA.

The sperm pellet was washed twice in cold TNE buffer at 2000 rpm for 7 min. The test started by adding 400 µL of cold acid-detergent solution (0.08 N HCl, 150 mM NaCl, 0.1% Triton-X 100, pH 1.2) to 200 µL of sperm suspension in TNE buffer. After exactly 30 s, 1.2 mL of cold working solution of acridine orange (0.6 mg of AO, 0.1 M citric acid, 0.2 M Na_2_PO_4_, 1 mM EDTA, 0.15 M NaCl, pH 6.0) was added to the sperm suspension. After 3 min, the samples were analyzed in a flow cytometer. Two subpopulations of sperm were identified: sperm with fragmented DNA emitting strong red fluorescence (DFI, DNA fragmentation index) and sperm with incompletely condensed chromatin emitting strong green fluorescence (HDS, High DNA Stainability). The percentage of both sperm subpopulations was statistically calculated [30].

#### 2.4.3. TUNEL Assay

The DNA fragmentation levels in sperm cells were determined by a direct method such as the TUNEL (Terminal deoxynucleotidyl transferase-mediated dUT nick-end labeling) assay using the FlowTACS Apoptosis Detection Kit (Trevigen, Inc. (Minneapolis, MN, USA). The principle of the method was the incorporation of labeled nucleotides into the free ends of 3′ DNA fragments in the presence of terminal deoxynucleotidyl transferase (TdT).

The previously fixed cells were washed in PBS at 2000 rpm for 5 min and permeabilized using 0.1% solution of Triton X-100 in 0.1% sodium citrate solution at 4 °C for 10 min. After washing in 1×Binding Buffer at 2000 rpm for 5 min, sperm pellets were resuspended in the reaction mixture containing 25 µL of 1 × Binding Buffer, 0.5 µL of biotinylated dNTP, 0.5 µL of Mn^2+^, and 0.5 µL of TdT enzyme and incubated at 37 °C for 45 min. The reaction was stopped by adding 1 mL of 1 × Stop Buffer. Next, the sperm pellet was resuspended in 25 µL of FITC-labeled streptavidin solution and incubated at room temperature for 20 min in the dark. To discriminate apoptotic cells from necrotic cells in the flow cytometry analysis, the sperm pellet was resuspended in PBS and incubated with 10 µL of propidium iodide (PI) at room temperature for 5 min. After cooling, the samples were analyzed in flow cytometry. Two sperm subpopulations were identified: sperm with DNA fragmentation (TUNEL-positive sperm) and sperm without DNA fragmentation (TUNEL-negative sperm). The percentage of TUNEL-positive sperm was statistically calculated. Additionally, two control samples were prepared: negative control for the level of background fluorescence (reaction mixture without TdT), and positive control for optimization of the labeling conditions (sperm pretreated with TACS-nuclease) [31].

#### 2.4.4. Aniline Blue (AB) Test

The AB test was used to assess sperm chromatin condensation abnormalities. This dye selectively binds to lysine residues (in histones). This test allows the identification of male gametes containing residual histones, which indicates abnormal spermiogenesis.

The previously fixed sperm smears were stained in 5% aqueous solution of aniline blue in 4% acetic acid (pH 3.5) for 5 min. After washing in distilled water, the slides were mounted with DPX. The smears were evaluated under a light microscope (DM 2000, Leica Microsystems, Wetzlar, Germany) under immersion at ×1000 magnification. A total of 200 spermatozoa in each preparation were counted. Two sperm subpopulations were identified: immature sperm with residual histones (dark blue/blue-stained head, AB-positive sperm) and sperm with mature chromatin without residual histones (pale blue-stained head, AB-negative sperm). The percentage of AB-positive sperm was calculated [32].

### 2.5. Statistical Analysis

Categorical data for the oxidative stress parameters were presented as numbers, while categorical data for the DNA/chromatin integrity parameters were presented as percentages. Descriptive statistics (median, range, mean, and standard deviation) were used to define continuous variables. The conformity of variables with normal distribution was evaluated using the Shapiro–Wilk test. As the variables were not normally distributed, the nonparametric Kruskal–Wallis test followed by the Dunn test with Holm correction was applied for comparative analysis of all the parameters among the studied groups. For assessment of correlations between oxidative stress and sperm chromatin/DNA integrity parameters, the Spearman rank test was used. All calculations were performed using the Python 3 with Pandas (https://pandas.pydata.org/ ver 0.24.2), Matplotlib (https://matplotlib.org/ ver 3.0.3), SciPy (https://www.scipy.org/ ver 1.2.1), Seaborn (https://seaborn.pydata.org/ ver 0.11.0) and scikit-posthoc (https://pypi.org/project/scikit-posthocs/ ver 0.5.4) libraries. A value of *p* < 0.05 was considered statistically significant for all analyses.

## 3. Results

### 3.1. Comparative Analysis of Oxidative Stress Parameters among the Studied Groups

The medians (range) and means with standard deviation values of seminal oxidative stress parameters in the studied groups of males are shown in Table 1. The total antioxidant capacity was significantly reduced only in the groups exposed to genital heat stress compared to the control group (*p* < 0.001 for professional drivers and *p* < 0.01 for infertile men with varicocele). Moreover, in these groups, the decline in TAC was accompanied by a significant increase in catalase activity compared to values obtained for the fertile group (*p* < 0.01 for professional drivers and *p* < 0.001 for infertile men with varicocele). The levels of seminal SOD activity and MDA concentration were similar in the groups under study, and no statistical differences were noted.

### 3.2. Comparative Analysis of Parameters for Sperm Chromatin/DNA Integrity among the Studied Groups of Males

**Figure 1:**
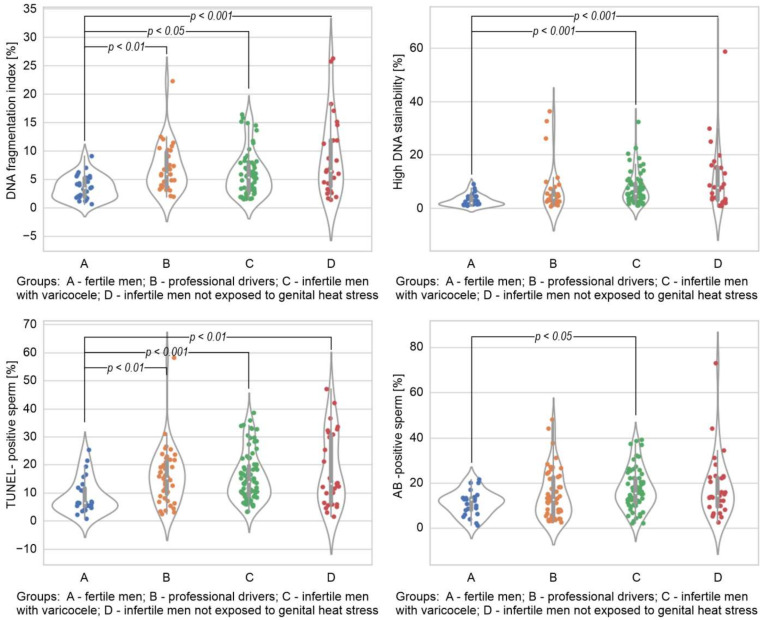
Comparison of sperm DNA fragmentation index, high DNA stainability index, and the percentage of TUNEL-positive and AB-positive sperm among the studied groups. The results are expressed as the median, Q1–Q3, and range. *p* < 0.05, *p* < 0.01, *p* < 0.001 calculated using the Dunn test with Holm’s correction compared to the control group.

The comparison of sperm DNA integrity parameters among the studied groups has been summarized in Figure 1. The DFI index measured in the SCSA was significantly higher in all the studied groups compared to the control (*p* < 0.05 for infertile men with varicocele; *p* < 0.01 for the group of drivers; and *p* < 0.001 for infertile men not exposed to genital heat stress). The HDS index measured in the SCSA was significantly higher in the infertile groups than the fertile men (*p* < 0.001). Similarly to the DFI index, the percentage of TUNEL-positive sperm cells was significantly higher in all the studied groups in respect of the values obtained in the group of fertile individuals (*p* < 0.01 for drivers and infertile men not exposed to genital heat stress and *p* < 0.001 for varicocele patients). A statistically significant difference was observed between the varicocele group and the fertile men in the percentage of sperm with immature chromatin in the AB test (*p* < 0.05).

### 3.3. Spearman Rank Order Correlations between Oxidative Stress and Sperm Chromatin/DNA Integrity Parameters in the Studied Groups of Males

**Figure 2:**
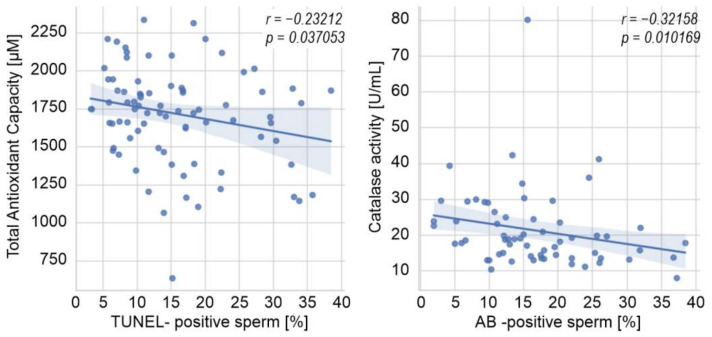
Spearman rank-order correlations between oxidative stress and sperm DNA integrity parameters in the group of infertile men with varicocele. AB—aniline blue, *p—p* value, *r—*Spearman’s correlation coefficient.

**Figure 3:**
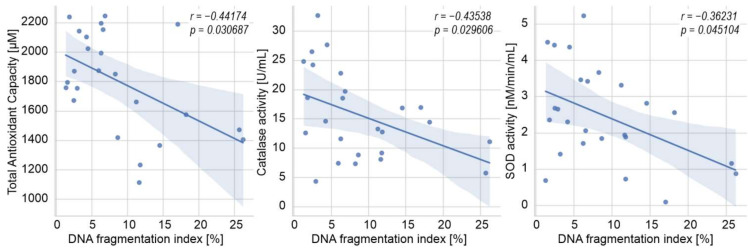
Spearman rank-order correlations between oxidative stress and sperm DNA integrity parameters in the group of infertile men not exposed to genital heat stress. *p—p* value, *r—*Spearman’s correlation coefficient, SOD—superoxide dismutase.

Figure 2 and Figure 3 show the Spearman rank order correlations between oxidative stress and sperm chromatin/DNA integrity parameters in the groups under study. In men with varicocele, a weak negative correlation was found between seminal TAC and the percentage of sperm cells with DNA fragmentation in the TUNEL assay (*p* < 0.05; Figure 2). Moreover, the decrease in catalase activity was associated with the increase in the percentage of AB-positive cells (*p* < 0.05; Figure 2). In the infertile men not exposed to genital heat stress, the DFI index was negatively correlated with TAC, catalase activity, and SOD activity (*p* < 0.05 for all cases; Figure 3). No statistically significant associations were found between the analyzed parameters in the group of drivers.

## 4. Discussion

It is widely accepted that oxidative stress is a common mechanism underlying the pathophysiology of male infertility, and is a risk factor in male fertility disorders. According to Esteves [33], over 80% of patients with fertility problems show oxidative stress in semen. As spermatozoa are characterized by an extremely high susceptibility to oxidative damage, seminal plasma released from accessory glands contains an abundant antioxidant system, which includes both enzymatic and nonenzymatic factors for effectively protecting spermatozoa against ROS attack. In this context, sperm cells are highly dependent on the powerful antioxidant properties of the surrounding environment [34]. Our previous report demonstrated that a redox imbalance in semen occurred in men exposed to prolonged environmental (professional drivers) or clinically recognized local hyperthermia (varicocele). Moreover, we have shown that the disturbances in the seminal oxidative stress scavenging system during active scrotal overheating can be principally associated with the metabolic disorders of the epididymis and prostate [25]. As we expected, in the present study, a greater number of semen samples in the studied subgroups not only allowed us to confirm the previous results but also to achieve an even higher statistical significance (Table 1). It seems that the low TAC in semen, despite an increased expression of some antioxidant enzymes, does not provide the proper protective effect on sperm cells from oxidative stress induced by heat stressors. Such data once again highlighted the importance of maintaining adequate levels of nonenzymatic antioxidant agents of seminal plasma to protect the sperm fertilizing potential. Analysis of research and clinical articles from the last 20 years revealed that antioxidant therapy was mostly investigated in male cohorts with sperm abnormalities, especially with reduced motility and clinical conditions such as idiopathic infertility and varicocele [35]. Taken together with the experimental findings showing a reduction in heat stress-induced oxidative stress in human sperm [36] or in TM4 Sertoli cells [37] in the presence of vitamin C, we can speculate that the administration of some nonenzymatic antioxidant molecules protects germ cells and/or sperm cells under conditions of genital heat stress. However, further research can answer the question whether this specific group of men can have any beneficial effects from such a therapeutic approach [4].

Regarding nonconventional seminological parameters, disagreements mostly concern the role of sperm chromatin/DNA integrity assessment [38,39,40]. The conflicting results were mainly associated with the methodology, variability in the studied population samples, the nature of sperm DNA breaks and their origin, and the prognostic significance of the determination of sperm nuclear chromatin/DNA integrity for infertility. In the present study, we applied the two most commonly used assays to measure the direct (TUNEL assay) and indirect (SCSA) levels of sperm DNA fragmentation. Additionally, disturbances in sperm chromatin condensation in the examined men were evaluated, directly as the population of AB-positive sperm cells with abnormal histone retention and indirectly as the population of sperm with incompletely condensed cells with high DNA stainability emitting strong green fluorescence in the SCSA. The conducted comparative analysis demonstrated a statistically significant increase in sperm DNA fragmentation levels in all the studied groups compared to the control. High medians of immature sperm cell numbers (in both tests) in the examined infertile subgroups, especially in varicocele patients, were also noted (Figure 1). Not surprisingly, we observed chromatin/DNA damage in men exposed as well as not exposed to prolonged heat stress because DNA fragmentation in sperm cells can be induced by a variety of pathological and environmental factors [41]. In addition, it is known to be prevalent among men with poor semen quality [42,43]. However, to the best of our knowledge, this is the first study documenting an elevated sperm DNA fragmentation in the group of professional drivers, suggesting a potential link between heat stress and levels of ejaculated sperm DNA fragmentation.

Apoptosis in ejaculated spermatozoa and its influence on the reproductive potential of male gametes has been a subject of considerable debate, especially in the context of the clinical significance of apoptotic markers and the postulated link between apoptosis and oxidative stress [13,14]. Under physiological conditions, the process of programmed cell death occurring in the seminiferous epithelium is a controlled and natural way of immature and damaged sperm elimination. On the other hand, it may be the cause of the deregulation of spermatogenesis and entry to various pathologies. There are substantial premises that apoptosis may be involved in the male gonad response to the thermogenic factor [2]. It was also evidenced by prospective experimental studies in humans in which transient scrotal hyperthermia demonstrated an increased expression of apoptotic markers, including caspase 3 activity and/or DNA fragmentation [44]. The role of apoptosis in the pathomechanism of infertility associated with varicocele was also strongly postulated in available reports [15,16,45]. Our results regarding the highest proportion of apoptotic TUNEL-positive sperm (propidium iodide was applied to discriminate apoptotic cells from necrotic cells) in varicocele patients are in agreement with the data obtained by other research groups, and further suggest a strong contribution of apoptosis in the pathogenesis of sperm DNA damage in clinically significant varicocele. Moreover, considering the elevated proportion of TUNEL-positive sperm in the group of professional drivers, we can speculate the occurrence of heat stress-induced apoptosis following the induction of ejaculated sperm DNA damage in this pathology. However, more studies including early and late apoptotic markers are needed to assess the involvement of sperm death processes in the male gonad response to heat stress of both internal and external origin.

This study was designed not only to compare oxidative stress intensity and sperm DNA damage in the groups of men exposed and not exposed to prolonged genital heat stress but also to provide associations among the parameters in the studied subgroups. Our results revealed such a direct relationship in the varicocele-positive infertile subjects (Figure 2), as well as in infertile men without active genital heat stress (Figure 3), showing some negative correlations among the studied antioxidant parameters (TAC, catalase, and/or SOD) and the percentage of sperm with DNA fragmentation. Such findings are consistent with other studies correlating the level of sperm DNA fragmentation with the activity of various antioxidant parameters in the semen of men with/without varicocele [46]. However, contrary to those studies, we were unable to find a direct connection between the lipid peroxidation measured by MDA concentration in seminal plasma and the level of sperm DNA fragmentation in any studied subgroups. This is not the first time that we have revealed a poor diagnostic value of seminal MDA concentration in male infertility [25].

Despite the fact that we demonstrated a clear co-existence of oxidative stress and sperm DNA fragmentation in ejaculates of professional drivers, the comparative analysis between the parameters did not show any direct relationship. However, it should be noted here that a lack of such significant correlations does not exclude the notion that heat-induced oxidative stress is associated with high levels of sperm DNA fragmentation in general. First, according to the current hypothesis, oxidative damage to sperm DNA naturally occurs, and it can be omitted in routine techniques used for the assessment of sperm DNA fragmentation [47]. Secondly, the potential adverse effects of heat-induced oxidative stress may be counteracted by the thermotolerance phenomenon that is generated in the testis after the first heat attack [48]. However, the induction of molecular mechanisms to protect germ cells against heat stressors (e.g., an antioxidant network within male gonads, the induction of a set of heat-shock proteins) facilitates cell survival; finally, it can induce responses to the chronic thermogenic factor in a different way, including variations in terms of epigenetics with a potential harmful impact on the reproductive success [49,50]. Thirdly, the detrimental effect of genital heat stress depends on the degree of temperature, its duration, and intervals between elevated temperature episodes. Fourthly, heat stress can be an additional factor triggering the consequences shown in this study in men with infertility.

Considering the increasing number of reports confirming the presence of seminal oxidative stress in men with varicocele, the simultaneous assessment of standard semen parameters, markers of sperm oxidation, and sperm chromatin/DNA integrity has become clinically significant in varicocele-related infertility. Moreover, the strategies to reduce sperm DNA damage with a focus on medical and surgical interventions in this pathology are the subject of intense debate [51,52]. It is noteworthy that the correlations revealed in the present study in the group of infertile men with varicocele notably indicated the participation of all the postulated mechanisms taking part in sperm chromatin/DNA failure. On the one hand, the observed negative correlation between the percentage of TUNEL-positive sperm and TAC levels indirectly supported the hypothesis about the oxidative origin of sperm fragmentation due to apoptotic process in the adult varicocele [53]. On the other hand, the negative correlation of the percentage of sperm with retained histones in the AB test with catalase activity determined the failure of the oxidative stress-induced maturation process in this pathology. Based on these data, it could be assumed that local oxidative stress in varicocele may play a central role in the pathogenesis of sperm DNA damage leading to the deregulation of spermatogenesis and spermiogenesis. We also cannot exclude that sperm maturation disturbances in this pathology could result from oxidative stress present in both the testis and epididymis [25].

Some relevant limitations of the present study should be mentioned. The group of infertile men with varicocele was not homogenous. The subjects with varicocele diagnosed as grade 1 were also included, which could weaken the strength of the correlations observed in this study group. However, the vast majority of patients had varicocele diagnosed as grade 2 or 3. Furthermore, the markers of sperm oxidative damage such as deoxyguanosine (8-OHdG), 4-hydroksynonenal (4-HNE), or mitochondrial ROS generation could have provided additional information regarding the considerable contribution of heat to the induction of oxidative stress in males exposed to external or internal hyperthermia.

Based on the evidence presented in the study, we can conclude that disturbances in the seminal oxidative stress scavenging system are engaged in the pathogenesis of sperm DNA damage triggering both spermatogenesis and spermiogenesis impairment. Local exposure to environmental factors associated with raised scrotal temperature can compromise the reproductive capacity in males due to the co-existence of sperm DNA fragmentation and oxidative stress in semen. Such data can clinically verify similar findings observed in the male germ line in the experimental animal models [54]. The conclusions presented, together with our previous data from the retrospective research study designed to assess the effect of thermogenic factors on ejaculated sperm alterations [25], also support a suggestion that the supplementation of exogenous antioxidants could be one of the therapeutic options to prevent or to reduce sperm damage under thermal conditions.

## Figures and Tables

**Table 1:** Descriptive statistics and comparative analysis of oxidative stress parameters among the studied groups of males.

Parameter	Fertile Men n = 30	Professional Drivers n = 61	Infertile Men with Varicocele n = 101	Infertile Men Not Exposed to Genital Heat Stress n = 40
Total antioxidant capacity (µM)	1916.50 (1583–2451) 1981.78 ± 247.82	1744 (890–2356) **b** 1686.7 ± 336.83	1770.50 (636–2459) **a** 1746.56 ± 336.58	1823.50 (1114–2246) 1810.66 ± 277.76
Catalase activity (U/mL)	12.63 (3.43–22.08) 12.45 ± 4.29	17.13(4.00–40.22) **a** 17.70 ± 7.55	17.52 (2.66–80.14) **b** 19.02 ± 10.47	16.01 (3.42 = 32.68) 16.24 ± 7.07
SOD activity (nM/min/mL)	2.70 (0.74–10.08) 2.76 ± 1.18	2.77 (0.36–8.48) 2.75 ± 1.07	2.68 (0.22–8.06) 2.75 ± 0.97	2.68 (0.15–8.23) 2.65 ± 1.22
MDA concentration (µM/mL)	3.16 (1.82–5.09) 3.22 ± 0.82	3.66 (1.71–7.21) 3.43 ± 1.18	3.12 (1.14–8.12) 3.25 ± 1.09	3.15 (1.01–5.30) 3.21 ± 0.97

Data are expressed as median (range) and mean ± SD; MDA—malondialdehyde; SOD—superoxide dismutase; **a** (*p* < 0.01), **b** (*p* < 0.001) calculated using Dunn test with Holm’s correction compared to the control group.

## Data Availability

The data presented in this study are available on request from the corresponding author (M.F.).
